# Measuring Statistical Asymmetries of Stochastic Processes: Study of the Autoregressive Process

**DOI:** 10.3390/e20070511

**Published:** 2018-07-07

**Authors:** Arthur Matsuo Yamashita Rios de Sousa, Hideki Takayasu, Misako Takayasu

**Affiliations:** 1 Department of Mathematical and Computing Science, School of Computing, Tokyo Institute of Technology, 4259, Nagatsuta-cho, Yokohama 226-8502, Japan; 2 Institute of Innovative Research, Tokyo Institute of Technology, 4259, Nagatsuta-cho, Yokohama 226-8502, Japan; 3 Sony Computer Science Laboratories, 3-14-13, Higashi-Gotanda, Shinagawa-ku, Tokyo 141-0022, Japan

**Keywords:** statistical symmetry, Kullback–Leibler divergence, stochastic process, autoregressive model, time series analysis

## Abstract

We use the definition of statistical symmetry as the invariance of a probability distribution under a given transformation and apply the concept to the underlying probability distribution of stochastic processes. To measure the degree of statistical asymmetry, we take the Kullback–Leibler divergence of a given probability distribution with respect to the corresponding transformed one and study it for the Gaussian autoregressive process using transformations on the temporal correlations’ structure. We then illustrate the employment of this notion as a time series analysis tool by measuring local statistical asymmetries of foreign exchange market price data for three transformations that capture distinct autocorrelation behaviors of the series—independence, non-negative correlations and Markovianity—obtaining a characterization of price movements in terms of each statistical symmetry.

## 1. Introduction

A basic definition of symmetry is invariance under transformation. The concept of symmetry is fundamental in mathematics, and it is readily evoked in Euclidean geometry, where a shape is symmetric if it remains unchanged after applying an operation such as a rotation about a point or a reflection with respect to a line [1].

The above definition of symmetry accounts for the exact invariance of an object under transformation. However, there are situations in which the transformed object is not exactly identical to the original on, but its statistical properties remain invariant. In this case, we qualify the symmetry as statistical. For instance, in the fractal geometry context, a deterministic fractal has the property of self-similarity associated with the scale symmetry: a fractal object is mapped to itself under an appropriate scale transformation. However, we can add stochastic elements in the construction of the object, and the scale symmetry is now statistical; it is a statistical fractal [2,3]. Naturally, fields using fractal models can also apply the concept of statistical (scale) symmetry, e.g., geosciences [4], atmospheric sciences [5] and physiology [6].

In previous studies, the term ‘statistical symmetry’ (or ‘average symmetry’) has been used to designate quantities that remain invariant on average [7,8,9]. Following [10], we use a more restrictive definition of statistical symmetry, requiring the invariance of the whole probability distribution under some transformation. Given a transformation *T*, we say that the probability distribution *P* is symmetric if the transformed distribution is equal to the original one:

(1) T[P]=P,

and an object governed by the probability distribution *P* has the statistical symmetry corresponding to the transformation *T*.

Considering a stochastic process as a mathematical object whose evolution is controlled by an underlying probability distribution, we can use statistical symmetries to characterize it. According to the type of symmetry we select, i.e., which transformation we use, information on different aspects of the process can be obtained.

Here, we propose the characterization of stochastic processes by measuring their degree of statistical asymmetry for certain transformations. We begin by specifying the statistical asymmetry measure as the Kullback–Leibler divergence of the probability distribution with respect to the transformed one, followed by the characterization of the Gaussian autoregressive process. Such a stochastic process is governed by a multivariate Gaussian distribution only specified by a covariance matrix and has a simple expression for the statistical asymmetry measure. Using this model as a linear approximation for the foreign exchange market price data, we exemplify the use of statistical symmetries to analyze real time series.

## 2. Measuring Statistical Asymmetry

Consider the transformation *T* on the probability distribution *P*, T[P], assumed to be also a probability distribution. If *P* is not symmetric with respect to *T*, then we have that T[P]≠P. We would like to have a quantity that reflects the degree of this asymmetry. As in [10], we find a suitable candidate in information theory, the Kullback–Leibler divergence (or relative entropy) [11,12,13,14]. The Kullback–Leibler divergence between *P* and T[P], with the possible events *X* in the sample space Ω, is given by:

(2) $$D\left(P||T\left[P\right]\right)=\sum_{X\in \Omega }P\left(X\right)\log\frac{P(X)}{T[P(X)]}.$$

The Kullback–Leibler divergence between *P* and T[P] can be understood as a measure of the amount of change in *P* after applying *T*, and then, D(P||T[P]) can be interpreted as a measure of asymmetry of *P* with respect to the transformation *T*. This quantity is only zero when T[P]=P, i.e., only for a symmetric distribution the measure of asymmetry is zero. Other measures of similarity between probability distributions exist, e.g., the Jensen–Shannon divergence [15,16,17], and they could also be used to measure statistical asymmetry. However, by using the Kullback–Leibler divergence we recover some quantities from information theory and statistical physics, as we exemplify next.

As a first example, we consider the total independence transformation, taking a probability distribution to the product of its marginal probability distributions. In the bivariate case, we have:

(3) $$T\left[P\left(x_{1},x_{2}\right)\right]=P\left(x_{1}\right)P\left(x_{2}\right).$$

Then:

(4) $$D\left(P||T\left[P\right]\right)=\sum_{x_{1},x_{2}}P\left(x_{1},x_{2}\right)\log\frac{P(x_{1},x_{2})}{P\left(x_{1}\right)P\left(x_{2}\right)},$$

which is the definition of the (bivariate) mutual information, measuring the dependence between the two random variables [12]. In the general case, for n arbitrary number of random variables, it coincides with the definition of multi-information (or total correlation) [18].

Another class of transformations is the one of space transformations, which assign a probability value of an event to another event of the sample space. For the vector of random variables $X={\left(x_{1},x_{2},\cdots ,x_{n}\right)}^{T}$, taking a transformation A, we write:

(5) T[P(X)]=P(AX).

For instance, the parity transformation takes $\left(x_{1},x_{2},\cdots ,x_{n}\right)$ to $\left(-x_{1},-x_{2},\cdots ,-x_{n}\right)$, and the probability distribution P(X) is symmetric if it is an even function.

We can also have permutations of $\left(x_{1},x_{2},\cdots ,x_{n}\right)$, with a symmetry associated with each possible permutation. One special permutation is the even reversion, transforming $\left(x_{1},x_{2},\cdots ,x_{n}\right)$ into $\left(x_{n},\cdots ,x_{2},x_{1}\right)$; combining it with the parity transformation, we obtain the odd reversion: $\left(x_{1},x_{2},\cdots ,x_{n}\right)\to \left(-x_{n},\cdots ,-x_{2},-x_{1}\right)$. The measure of statistical asymmetry for reversion is:

(6) $$D\left(P||T\left[P\right]\right)=\sum_{x_{1},x_{2},\cdots ,x_{n}}P\left(x_{1},x_{2},\cdots ,x_{n}\right)\log\frac{P(x_{1},x_{2},\cdots ,x_{n})}{P(\pm x_{n},\cdots ,\pm x_{2},\pm x_{1})},$$

with a plus sign if the transformation is an even reversion and a minus sign if it is an odd reversion.

If we are studying the time evolution of an even (odd) variable *x*, i.e., if the indexes in the path $\left(x_{1},x_{2},\cdots ,x_{n}\right)$ represent time, the symmetry with respect to even (odd) reversion transformations can be regarded as time reversibility, and the measure of statistical asymmetry D(P||T[P]) quantifies the degree of time irreversibility of the considered temporal path. This type of symmetry is used in the area of non-equilibrium physics, with D(P||T[P]) being closely related to the notion of entropy production [19,20,21].

## 3. Statistical Symmetries of the Gaussian Autoregressive Process

Having defined the measure of statistical asymmetry, we proceed to the characterization of stochastic processes. We specifically study statistical symmetries of the autoregressive process of order *n* with Gaussian error term defined by:

(7) $$x_{t}=\sum_{j=1}^{n}\phi_{j}x_{t-j}+\xi_{t},$$

with $\phi_{j}$ being constant real coefficients and $\xi_{t}$ independent Gaussian random variables with mean zero and variance $\sigma^{2}$. Besides its simplicity, the study of the autoregressive process is justified by its importance in time series modeling [22,23,24].

We consider the stationary autoregressive model, with all the roots of the characteristic polynomial $p\left(\lambda \right)=\lambda^{n}-\sum_{j=1}^{n}\phi_{j}\lambda^{n-j}$ inside the unit circle in the complex plane. For large *t*, the vector $X=(x_{t},x_{t-1},\cdots ,x_{t-m})$ follows a multivariate Gaussian distribution with null mean vector [25]:

(8) $$f\left(X\right)=\frac{1}{\sqrt{{\left(2\pi \right)}^{m+1}det\left(C\right)}}\exp\left(-\frac{1}{2}X^{T}C^{-1}X\right),$$

where C is the covariance matrix related to vector X. Observe that for the stationary Gaussian autoregressive model, the covariance matrix is a symmetric Toeplitz matrix.

We can then investigate the symmetries (i.e., invariance under transformations) of this multivariate Gaussian distribution and measure the statistical asymmetries of a temporal path X of the process. Let us take only transformations mapping the original distribution to another multivariate Gaussian distribution, so that the Kullback–Leibler divergence *D* only depends on the covariance matrices [26]:

(9) $$D\equiv D\left(f||T\left[f\right]\right)=\frac{1}{2}\left[\ln\frac{det(\tilde{C})}{det(C)}+tr\left({\tilde{C}}^{-1}C\right)-\left(m+1\right)\right],$$

where $\tilde{C}$ is the covariance matrix of the transformed Gaussian distribution, ln is the natural logarithm and *D* is measured in nats.

Considering space transformations, observe that a path X from a stationary Gaussian autoregressive process is always statistically symmetric with respect to parity and reversion transformations, so this process is always time reversible for even or odd variables [27,28]; time irreversibility arises when non-stationarity is present.

Instead of space transformations, we explore transformations acting directly on the covariance matrix. We choose three transformations on the covariance matrix: the total independence transformation, the non-negative covariance transformation and the geometric covariance transformation:

1. The total independence transformation keeps the elements of the principal diagonal and transforms the elements $c_{jk}$, j≠k, of the covariance matrix into zeros:
  (10) $${\tilde{c}}_{jk\neq j}=0;$$
  The independent process (white noise) $x_{t}=\xi_{t}$, not depending on past values, has the total independence statistical symmetry.
2. The non-negative covariance transformation makes:
  (11) $${\tilde{c}}_{jk}=\left|c_{jk}\right|,$$
  i.e., it makes all the elements of the covariance matrix non-negative; the symmetric processes are the ones with only non-negatively correlated variables.
3. The geometric covariance transformation produces a geometric decay in the covariance:
  (12) $${\tilde{c}}_{jk}=c_{jj}{\left(\frac{c_{j(j+1)}}{c_{jj}}\right)}^{\left|j-k\right|},$$
  which is the covariance decay behavior of autoregressive processes of order n=1, being Markovian and the symmetric processes for this transformation. Note that the independent process is also statistically symmetric with respect to the geometric and non-negative covariance transformations.

We estimate the statistical asymmetry measure *D* for each one of the three previous transformations for a simulated Gaussian autoregressive process with parameters changing every 10,000 steps, but keeping unit variance (see Table 1).

**Figure 1:**
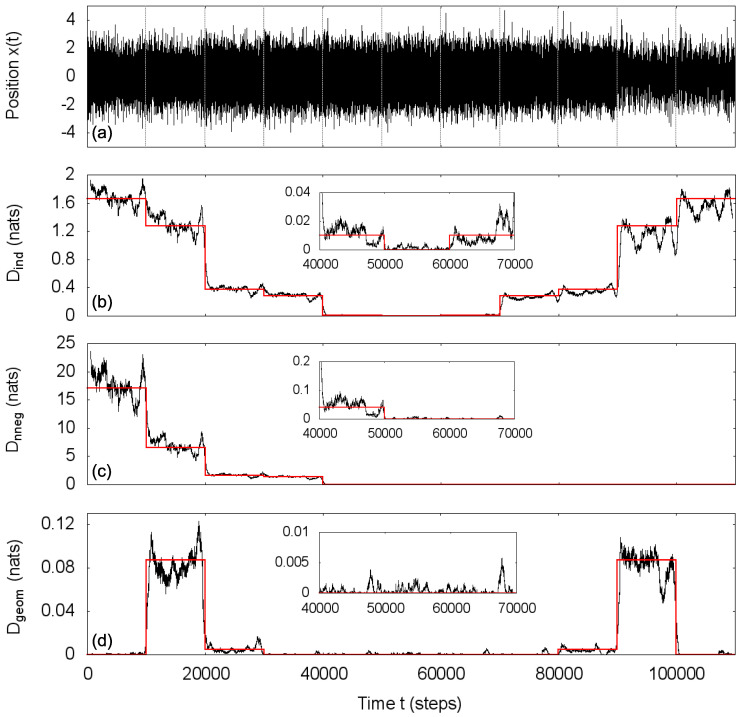
(**a**) Simulated Gaussian autoregressive process with the parameters specified in Table 1 and estimations of the statistical asymmetry measure *D* for the transformations: (**b**) total independence transformation, (**c**) non-negative covariance transformation and (**d**) geometric covariance transformation, using path length m+1=3 and window size w=1000. Red lines are the theoretical values, and D=0 indicates intervals when the process is statistically symmetric for the considered transformation.

Intending a local analysis, we utilize a sliding window procedure. In each window of size *w*, regarded as stationary, we estimate the covariance matrix of a path $\left(x_{t},x_{t-1},\cdots ,x_{t-m}\right)$ and compute the measure *D* using Equation (9) for each one of the studied transformations: $D_{ind}$ for total independence transformation (Equation (10)), $D_{nneg}$ for non-negative covariance transformation (Equation (11)) and $D_{geom}$ for geometric covariance transformation (Equation (12)). As in any such procedure, the size *w* of the window should be large enough for a reasonable estimation of the covariances and small enough for a local characterization. In Figure 1, we show the results for w=1000 and m=2; red lines correspond to the exact values of the statistical asymmetry measures, where covariances were computed using the Yule–Walker equations [24] (transitions between different sets of parameters were ignored).

In this simulated process, the estimations of statistical asymmetry measures closely follow the theoretical values and they are able to distinguish the distinct behaviors associated with each of the considered transformations: for the total independence transformation, $D_{ind}$ is minimum (≈0) for the independent process ($\phi_{1}=\phi_{2}=0$) and increases as the parameters distance from zero; for the non-negative covariance transformation, $D_{nneg}\approx 0$ identifies the processes whose autocorrelation function is always non-negative; and for the geometric covariance transformation, the estimations of $D_{geom}$ that largely deviates from zero indicate the processes that are not Markovian, in this case autoregressive processes of order n=2 ($\phi_{2}\neq 0$). Note that the transformations were conveniently chosen so that the statistically symmetric Gaussian autoregressive processes come from three (not mutually exclusive) classes according to their autocorrelation structure—independent, non-negative correlations, Markovian—and *D* measures the deviation of the actual process from each of those classes.

**Figure 2:**
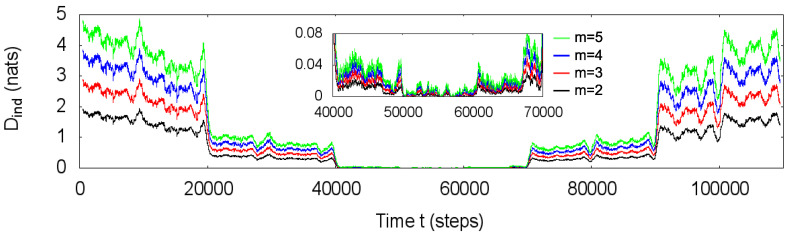
For the same process in Figure 1a, estimations of the statistical asymmetry measure *D* for the total independence transformation considering paths $\left(x_{t},x_{t-1},\cdots ,x_{t-m}\right)$ with length m=2,3,4 and 5. An increase in the value of *m* increases the magnitude of *D*, but keeps the relative degree of statistical asymmetry along the process.

The path length m+1=3 was chosen because the simulated process includes only autoregressive processes of order n≤2, and the effects of the three described transformations are already appreciable for this value of *m*. Analyzing the same simulated process, Figure 2 presents the estimations of *D* for the total independence transformation using m=2,3,4 and 5, evidencing that for this process, the increase of the value of *m* only changes the magnitude of *D* and does not interfere in the characterization of the process in terms of the relative degree of statistical asymmetry. Indeed, for general Gaussian autoregressive processes, a value of *m* greater than the order *n* is over-informative since the parameters defining the process are fully determined by its variance and the *n* first lags of the autocovariance function (Yule–Walker equations). The choice of the value of *m* is also restricted by the utilized transformations (e.g., the geometric covariance transformation requires m≥2 to be useful) and, in practice, by the statistical significance of the estimations of the covariances.

**Figure 3:**
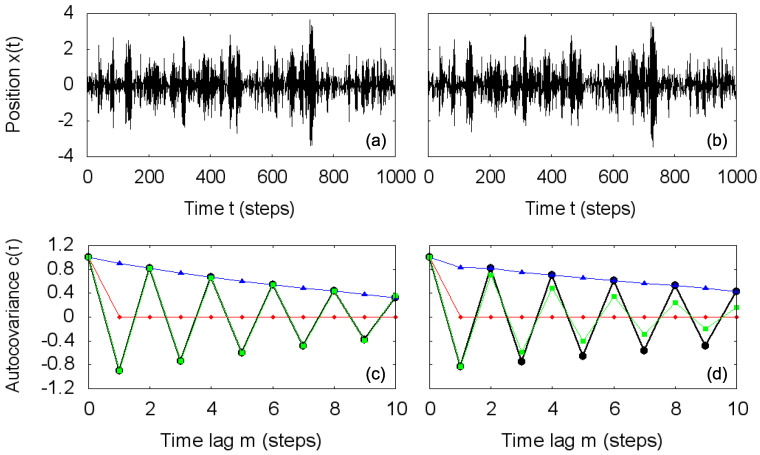
Simulated Gaussian autoregressive process with parameters: (**a**) $\phi_{1}=-0.9$, $\phi_{2}=0$, σ=0.436 and (**b**) $\phi_{1}=-0.5$, $\phi_{2}=0.4$, σ=0.507. (**c,d**) Autocovariance function for the simulated processes (black circles) and its transformed versions for total independence transformation (red diamonds), non-negative covariance transformation (blue triangles) and geometric covariance transformation (green squares).

A more detailed view of the transformations on the covariance matrix associated with a Gaussian autoregressive process is exhibited in Figure 3. Using the same set of random numbers for a direct comparison, we simulate two autoregressive processes with the parameters in the first two rows of Table 1: $\phi_{1}=-0.9$, $\phi_{2}=0$, σ=0.436 and $\phi_{1}=-0.5$, $\phi_{2}=0.4$, σ=0.507 (Figure 3a,b, respectively), visually presenting similar behavior. Figure 3c,d displays the corresponding estimated autocovariance functions (black circles) and the results of the application of the total independence transformation (Equation (10), red diamonds), the non-negative covariance transformation (Equation (11), blue triangles) and the geometric covariance transformation (Equation (12), green squares). Simply from the functional form of the autocovariance functions, with negative values of covariances, we readily observe deviations from the independent and non-negatively correlated processes. For the geometric covariance transformation, visual inspection is not enough, and only a comparison between the autocovariance function and the transformed one reveals that while the first process is Markovian, the second one does not have covariances compatible with an autoregressive process of order n=1, being non-Markovian. The quantities $D_{ind}$, $D_{nneg}$ and $D_{geom}$ measure those deviations and are particularly useful in local analysis, where the inspection of the whole autocovariance (or autocorrelation) function in each window is not practicable.

## 4. Application to Market Price Time Series Data

In order to exemplify how the concept of statistical symmetry can be applied to analyze time series data, we characterize price time series from the foreign exchange market by measuring its local statistical asymmetries. The dataset used here was purchased from the Electronic Broking Service (EBS) and contains traders’ quotes (desired transaction prices) for buying or selling an amount of a currency, with the mid-quote defined at each time as the average of the best quote from the buy side and from the sell side. We focus on the pair U.S. dollar (USD) and Japanese yen (JPY) and analyze the mid-quote time series with 1 s resolution for two days: 5 December 2011, representing an ordinary day, and 4 August 2011, an atypical day when there was an intervention in the market by the Japanese government.

The use of Equation (9) to measure statistical asymmetry and the interpretations of the previously presented transformations presuppose modeling the time series as a Gaussian autoregressive process. The market price dynamics, however, is usually not Gaussian; in fact, price changes commonly follow a heavy-tailed distributions [29]. Nevertheless, the Gaussian autoregressive model continues to be utilized as a linear approximation for price changes, being simple enough to derive analytical results and serving as an ingredient for more elaborated models [30,31,32]. Other than that, standard models for price changes are based on random coefficient autoregressive processes, centered on the autoregressive conditional heteroskedasticity (ARCH) model, which reproduce the heavy-tailed distribution and maintain the covariance matrix (up to a multiplicative factor) of the ordinary constant coefficient autoregressive process [33,34,35]. Thus, since our analysis focuses on the autocovariances of the price time series, the Gaussian approximation is justified, but knowing that we are actually analyzing the Gaussian version of the time series, i.e., the Gaussian process producing the same temporal correlation structure observed in the original series. Observe that the measure of statistical asymmetries as we propose does not require the explicit estimation of the model parameters, but only the covariance matrix; in fact, in a general case, it does not even require the specification of the model provided it is Gaussian.

**Figure 4:**
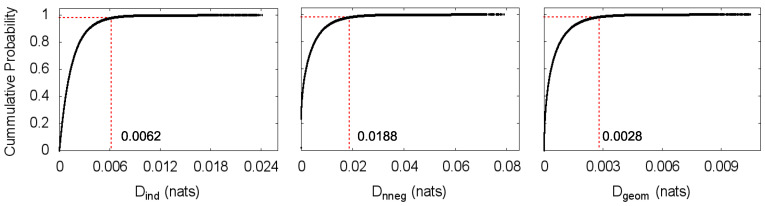
Estimated cumulative probability distributions of *D* for the independent process and the transformations: (**a**) total independence transformation; (**b**) non-negative covariance transformation; and (**c**) geometric covariance transformation. Red dashed lines indicate the values of *D* whose cumulative probability is 0.98, $D_{ind}=0.0062$, $D_{nneg}=0.0188$ and $D_{geom}=0.0028$, respectively.

**Figure 5:**
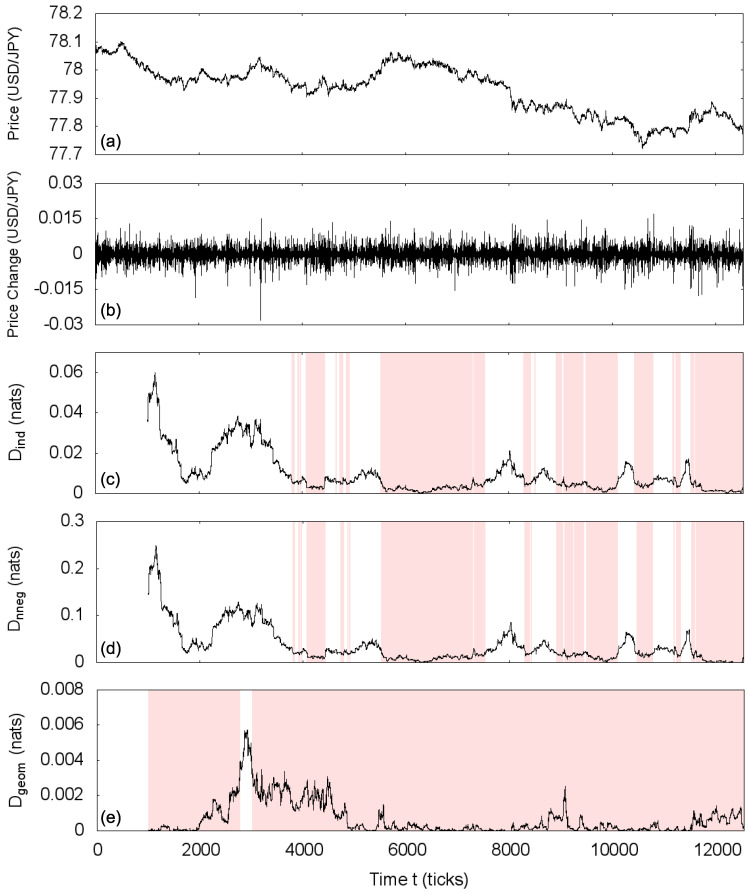
(**a**) Time series of the mid-quote of USD/JPY for 5 December 2011; (**b**) time series of price changes for the same day and estimations of the statistical asymmetry measure *D* for the transformations: (**c**) Total independence transformation, (**d**) non-negative covariance transformation and (**e**) geometric covariance transformation, using path length m=2 and window size w=1000. Shaded areas indicate intervals classified as statistically symmetric.

We perform the same local statistical asymmetry analysis described in the previous section with path length m+1=3 and window size w=1000 on the market mid-quote (price) changes time series, which are expressed in tick time, i.e., the time steps with no price change are removed. For these data, we label a value *D* with the time corresponding to the end of the window it was computed in, so that this value refers only to previous steps. As seen for the simulated process, the computed statistical asymmetry measure may not be exactly zero for the symmetric processes due to an imperfect estimation of the covariance matrix. We then use the value below which lie 98% of the estimations of *D* for the independent process as the threshold for an interval to be classified as statistically asymmetric. From the estimated cumulative probability distributions of *D* for the independent process, this value is $D_{ind}=0.0062$ for the total independence transformation, $D_{nneg}=0.0188$ for the non-negative covariance transformation and $D_{geom}=0.0028$ for the geometric covariance transformation (Figure 4). Results are displayed in Figure 5, in which shaded areas designate statistically symmetric intervals.

The USD/JPY market on 5 December 2011 presents only mild volatility and no major events, but its temporal correlations are not homogeneous. Besides locating the statistically symmetric intervals, this analysis enables us to observe the evolution of the relative degree of asymmetry of the USD/JPY market along the day. First, for the total independence statistical symmetry, at the beginning of the day, the price change time series presents important deviations from the symmetric process and cannot be characterized as independent, but shows statistically independent intervals after this initial period (Figure 5c); we highlight that 5 December 2011 is a Monday, when the market is reopened after the weekend, possibly explaining the initial relatively large asymmetric behavior of price changes. Then, we notice that the non-negative covariance statically symmetric intervals coincide with the independent ones (Figure 5d); of course, the independent symmetry implies non-negative covariance symmetry, but it is possible that non-negative covariance symmetry exists in independent asymmetric intervals (compare Figure 1c,d). The interpretation is that the price changes in this day are either non-correlated or present important negative correlations; such observation agrees with the stylized fact that the autocorrelation of price changes goes quickly to zero after a possible short negative correlation [36]. We also note that the evolutions of $D_{ind}$ and $D_{nneg}$ are very similar (up to the difference in scales); this behavior is not unexpected since in this case, the positive-negative zig-zag of the autocovariance function leading to a high value of $D_{nneg}$ implies a high value of $D_{ind}$ as well. Finally, the analyzed day is essentially geometric covariance statistically symmetric (Figure 5e) (with the exception of a short interval at the beginning of the day), being described by a Markov process, also consistent with the known rapid decay of the autocorrelation function of market price changes.

**Figure 6:**
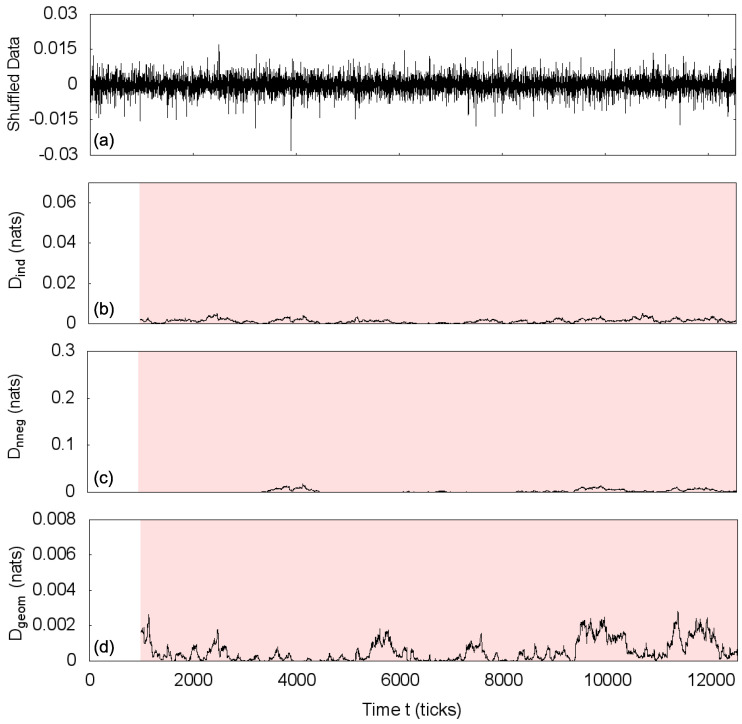
(**a**) Shuffling of time series of price changes for 5 December 2011 and estimations of the statistical asymmetry measure *D* for the transformations: (**b**) Total independence transformation, (**c**) non-negative covariance transformation and (**d**) geometric covariance transformation, using path length m=2 and window size w=1000. Shaded areas indicate intervals classified as statistically symmetric. The whole shuffled time series is evaluated as statistically symmetric for all three transformations.

Next, we randomize the market price changes data of 5 December 2011 and apply the same methodology. Figure 6 shows that the shuffled time series presents the same correlation structure of the independent process, being totally statistically symmetric for all three studied transformations and confirming that the detected asymmetries in the original time series indeed reveal information about the intrinsic correlations in the market dynamics.

**Figure 7:**
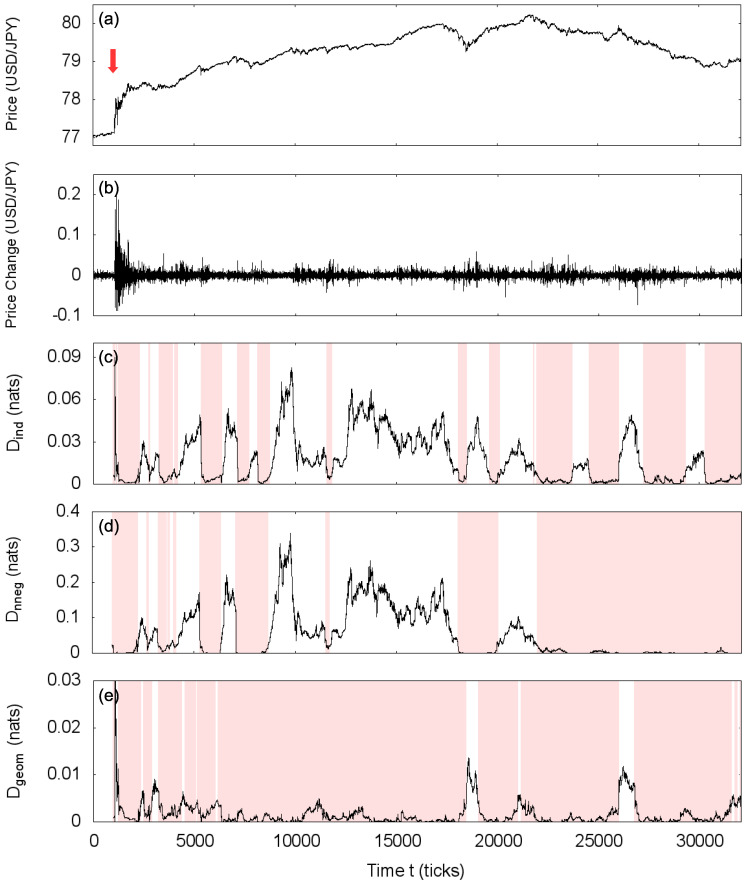
(**a**) Time series of the mid-quote of USD/JPY for 4 August 2011 (red arrow indicates the Japanese government intervention); (**b**) time series of price changes for the same day and estimations of the statistical asymmetry measure *D* for the transformations; (**c**) Total independence transformation; (**d**) non-negative covariance transformation and (**e**) geometric covariance transformation, using path length m=2 and window size w=1000. Shaded areas indicate intervals classified as statistically symmetric.

At last, we analyze the USD/JPY market on 4 August 2011 (Thursday). On that day, as the Japan economy was still recovering from the earthquake of March 2011, the Japanese government intervened in the market selling Yen to weaken the currency and stimulate exports [37]. Although the Gaussian approximation is dubious in windows around this significant event, we can still compute the statistical symmetries and study the price correlation structure of periods after the intervention. Results are shown in Figure 7, with important differences when compared to the characterization of 5 December 2011 (Figure 5). Firstly, the scales: in the intervals classified as statistically asymmetric, the degree of asymmetry is in general higher than in the asymmetric intervals of the ordinary day. Secondly, we can find non-negative covariance statistically symmetric intervals that are not independently symmetric (compare Figure 7c,d), indicating positive correlations. Lastly, the presence of a few windows presenting high geometric covariance asymmetry, not manifesting the Markov property (the two prominent ones around t≈ 18,000 and t≈ 26,000 in Figure 7e). A systematic investigation is still needed to evaluate if those are general differences between typical days and periods with major events on the market or if they are particular for each day.

In the context of financial data, the presented statistical symmetry analysis using transformations on the covariances can be applied, for instance in real-time characterization of markets, providing a practical monitoring tool for practitioners and allowing them to adjust their strategies according to changes in the correlation state of the market.

## 5. Final Remarks

In this work, we studied a specific definition of statistical symmetry that deals directly with the underlying probability distribution governing some system. Defining it this way, we could use the Kullback–Leibler divergence to measure the degree of statistical asymmetry with respect to a given transformation. We focused on statistical symmetries of a simple stochastic process, the Gaussian autoregressive process, which follows a multivariate Gaussian distribution and then allows us to express the statistical asymmetry measure in terms of its covariance matrix. Three transformations were selected to characterize the Gaussian autoregressive process, each corresponding to different aspects of the autocorrelation of the process: total independence transformation (independence), non-negative covariance transformation (non-negative correlations) and geometric covariance transformation (Markovianity).

We provided an example of time series analysis using statistical symmetries by considering Gaussian autoregressive processes that reproduce the autocovariances of the USD/JPY market price change dynamics and measuring its local statistical asymmetries through a sliding window procedure. We not only obtained the identification of statistically symmetric intervals (and thus, their properties of independence, non-negative correlations and Markovianity), but also the relative degree of statistical asymmetry for different periods of the day, revealing the evolution of the price correlation structure.

For the three examined transformations on the Gaussian autoregressive process, the statistical asymmetry measure acts as a single information theoretically-based index for the temporal correlations’ structure of the process and stands as a potential tool for the characterization of time series that can be approximated by such a model. For data not admitting a Gaussian description, one can estimate the underlying probability distribution of the time series and compute measures of statistical asymmetry using that estimation. A more general analysis framework can be achieved with the study of other stochastic models and defining useful transformations to investigate different aspects of a given process.

## Figures and Tables

**Table 1:** Parameters of the simulated Gaussian autoregressive process.

Interval (Steps)	$\phi_{1}$	$\phi_{2}$	σ
1–10,000	−0.9	0	0.436
10,001–20,000	−0.5	0.4	0.507
20,001–30,000	−0.5	0.1	0.827
30,001–40,000	−0.5	0	0.866
40,001–50,000	−0.1	0	0.995
50,001–60,000	0	0	1
60,001–70,000	0.1	0	0.995
70,001–80,000	0.5	0	0.866
80,001–90,000	0.5	0.1	0.827
90,001–100,000	0.5	0.4	0.507
100,001–110,000	0.9	0	0.436
